# Recurrent benign fibrous histiocytoma of the bone benefits from denosumab followed by malignant transformation: a case report

**DOI:** 10.1007/s00256-024-04610-w

**Published:** 2024-02-19

**Authors:** Wetterwald Laureline, Omoumi Patrick, Nguyen Tu, Cherix Stephane, Dolcan Ana, Ferraro Daniela, Saglietti Chiara, Letovanec Igor, Digklia Antonia

**Affiliations:** 1 Department of Oncology, Centre Hospitalier Universitaire Vaudois and University of Lausanne, Lausanne, Switzerland; 2 Department of Diagnostic Radiology And Interventional Radiology, Lausanne University Hospital and University of Lausanne, Lausanne, Switzerland; 3 Department of Orthopaedics and Traumatology, Lausanne University Hospital, Lausanne, Switzerland; 4 Institute of Pathology, Lausanne University Hospital and University of Lausanne, Lausanne, Switzerland

**Keywords:** Benign fibrous histiocytoma of bone (BFHB), Denosumab, Pelvic bone, Transformation, Cancer

## Abstract

Benign fibrous histiocytoma of the bone (BFHB) is a rare mesenchymal tumor, representing less than 1% of all benign bone tumors. This controversial entity is characterized by a mixture of fibroblasts arranged in a storiform pattern, varying amounts of osteoclast-type giant cells and foamy macrophages. Curettage or simple resection is usually curative. However, it was reported that up to 11% of the patients suffer from relapse. Here, we report a case of malignant transformation of BFHB after long-lasting disease stabilization under denosumab therapy.

## Introduction

Benign fibrous histiocytoma (BFH) is a very rare entity first described by Stout and Lates in 1967 [1]. Primary bone involvement is rare, with only about 100 total reported cases, representing less than 1% of all benign bone tumors. BFH of bone (BFHB) usually arises the pelvic and long bones, especially the femur and tibia [2]. As for its 2020 version, the WHO classification of bone and soft tissue tumors challenges the individual entity of BFHB, describing it as a giant cell tumor of bone (GCTB) with regressive changes [3].

Clinically, BFHB may be asymptomatic but usually presents with local pain and, depending on the locations, symptoms related to compression or involvement of adjacent structures and organs. Pathologic fracture is exceptional [4]. Radiologically, BFHB appears as a well-defined lytic lesion with a sclerotic margin. It may have a loculated appearance with internal trabeculations [5]. Histologically, BFHB is characterized by spindle-shaped fibrohistiocytic cells arranged in storiform pattern. Lipid-filled cells, foam cells, and multinucleated osteoclast-type giant cells are frequently found scattered among the stroma [6]. Differential diagnosis includes non-ossifying fibroma (NOF) and GCTB. NOF is histologically indistinguishable from BFHB but differs by its sites of skeletal involvement, typically the metaphysis of long bones, and affects younger patients. GCT with extensive areas of fibrocystic reaction might be misdiagnosed as BFHB. In these cases, the involvement of the epiphysis of a long bone should strengthen the diagnosis of GCTB. Therefore, BFHB diagnosis should be made by exclusion of those other entities.

Currently, there are no treatment guidelines for BFHB. Curettage or surgical excision is usually curative. While it has been reported that up to 11% BFHB can recur locally if not completely excised [7], malignant change is exceptional [8, 9].

## Case report

**Fig. 1:**
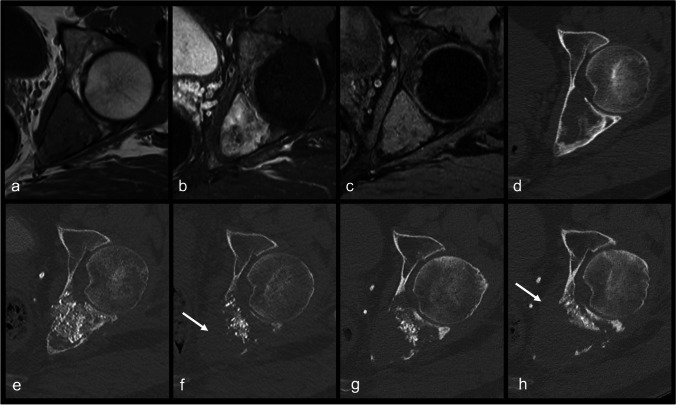
Imaging of the bone lesion at different stages of treatment. MR images (**a**, **b**, **c**) show a tumor located in the posterior wall of the acetabulum, which is isointense to muscle on T1-weighted sequences (**a**), hyperintense with slight heterogeneity on fat-suppressed T2-weighted sequences (**b**), and moderate enhancement on fat-suppressed post-contrast T1-weighted sequences (**c**). On CT (**d**), the lesion is well-defined, osteolytic, with a rim of sclerosis at the periphery and areas of cortical scalloping. Post-operative CT after curettage is shown (**e**). Three years after treatment, CT (**f**) shows diffuse bone lysis, as well as progression into soft tissues (arrow). One year after initiation of denosumab therapy, CT (**g**) shows a decrease in the tumor size and ossification notably close to the subchondral bone. About 2 years of the initiation of denosumab, CT (**h**) shows signs of tumor progression, with soft tissue extension (arrow) as well as bone lysis

**Fig. 2:**
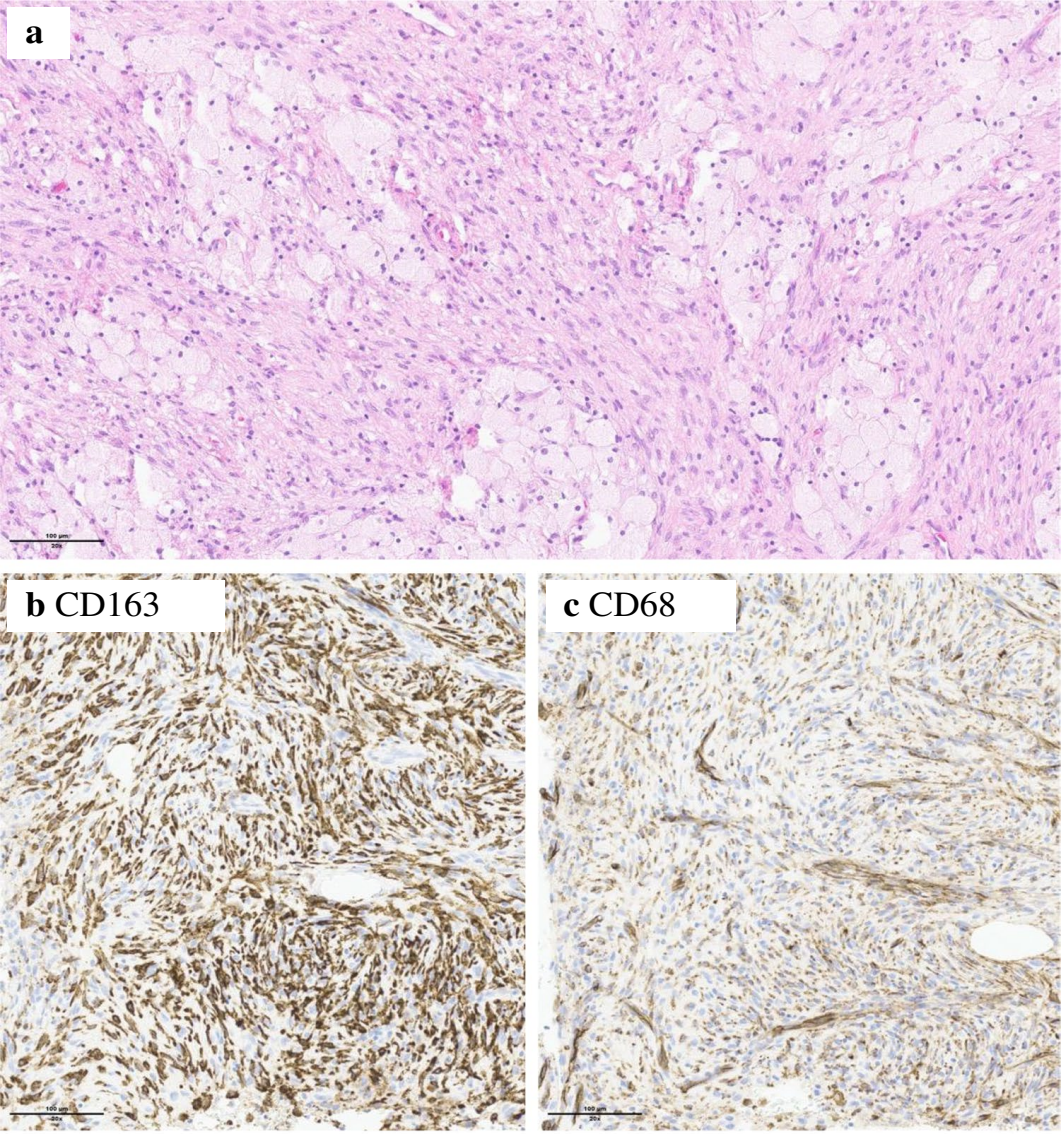
On surgical biopsy, the lesion was composed by a loose proliferation of spindle cells accompanied by numerous clusters of abundant, lipid-laden macrophages (**a** magnification × 10, scale bar 200 µm). The spindle cells expressed CD163 (**b** magnification × 20, scale bar 100 µm) and CD68 (**c** magnification × 20, scale bar 100 µm)

A 44-year-old man presented to our institution because of persisting left hip pain after a fall. A lytic mass affecting the left hemipelvis was found (Fig. 1a–d). Surgical biopsy was performed. Histologically, the lesion was composed of spindle-shaped cells growing in storiform pattern with large clusters of lipid-laden histiocytes and rare osteoclast-type giant cells. There were no features of malignancy such as increased mitotic index or the presence of necrosis. The cells were positive for CD163 and CD68. SMA and S-100 were not expressed. These morphologic features along with the absence of GNAS mutation were consistent with the diagnosis of a benign fibrous histiocytoma of bone (BFHB) with extensive xanthomatous changes (Fig. 2).

**Fig. 3:**
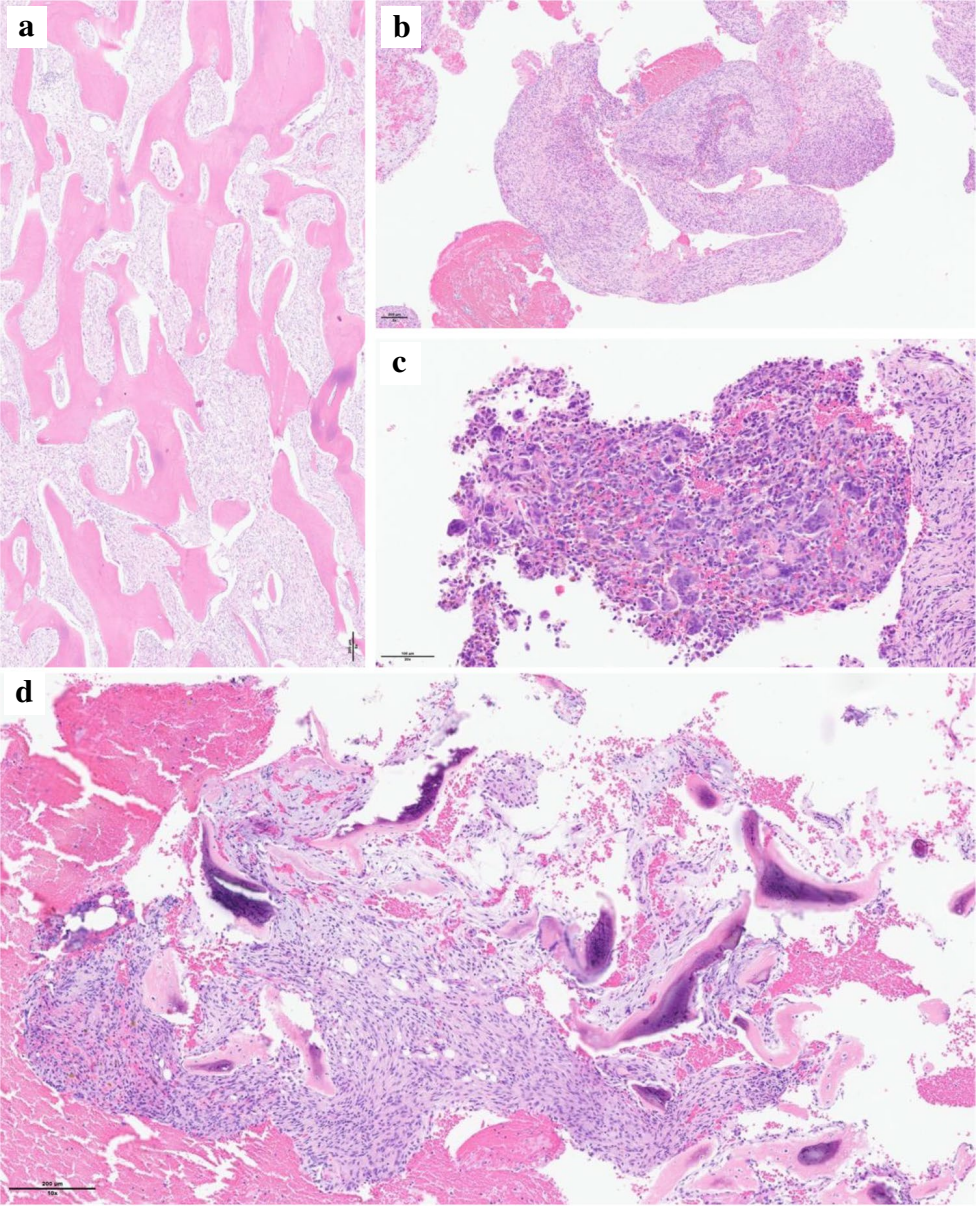
The first relapse was histologically characterized by fibrous area with new bone formation (**a** magnification × 5, scale bar 200 µm). Aneurysmal cyst-like areas showing a cyst wall composed of spindle cells (**b** magnification × 5, scale bar 200 µm) with scattered osteoclast-like giant cells and hemosiderin pigment deposition in the presence of hemorrhage (**c** magnification × 20, scale bar 200 µm). A non-atypical spindle cell proliferation was observed in the second relapse biopsy, which was similar in appearance to that previously observed (**d** magnification × 10, scale bar 200 µm)

The patient was treated by curettage and bone grafting and required a second curettage 2 years later after a first local relapse. The specimen showed more fibrous areas with bone formation and aneurysmal bone cyst–like areas reminiscent of a fibrous dysplasia with secondary changes (Fig. 3a–c). PCR for *GNAS* mutation was repeated and came negative again.

Three years after initial surgical treatment, the patient presented with increasing hip pain and loading impairment. The radiologic workup by contrast-enhanced magnetic resonance imaging (MRI) and computed tomography (CT) revealed disease progression with areas of cortical lysis and invasion of adjacent soft tissues (Fig. 1f). The fluorodeoxyglucose (FDG)-positron emission tomography (PET)/CT showed a hypermetabolic lesion and excluded the presence of metastasis. The histopathologic features of this second relapse corresponded to a BFHB, as observed in the previous specimens, without any sign of malignant transformation (Fig. 3d). The surgical option being internal hemipelvectomy, we performed radiotherapy with irradiation of the left hemipelvis at a total dose of 56 Gy, in 28 fractions [10]. Unfortunately, 1 month after radiation therapy, the patient described increased pain, and radiologic evaluation showed ongoing disease progression.

We proposed off-label use of denosumab 120 mg monthly, aiming at decreasing the osteolytic portion thus reducing the risk a fracture. The patient accepted our proposal and began the treatment in May 2020. After 2 months, he reported improved pain. Furthermore, CT revealed a decrease in size of the osteolytic areas as well as signs of ossification. Over a 2-year period, the patient displayed clinical benefits, including a reduction in pain requiring less analgesia, and improvements in function and mobility, with good treatment compliance. Additionally, for 14 months, the radiologic follow-up showed the decrease in size of the osteolytic lesion and increased ossification (Fig. 1g). Due to the clinical benefit, we continued the treatment.

**Fig. 4:**
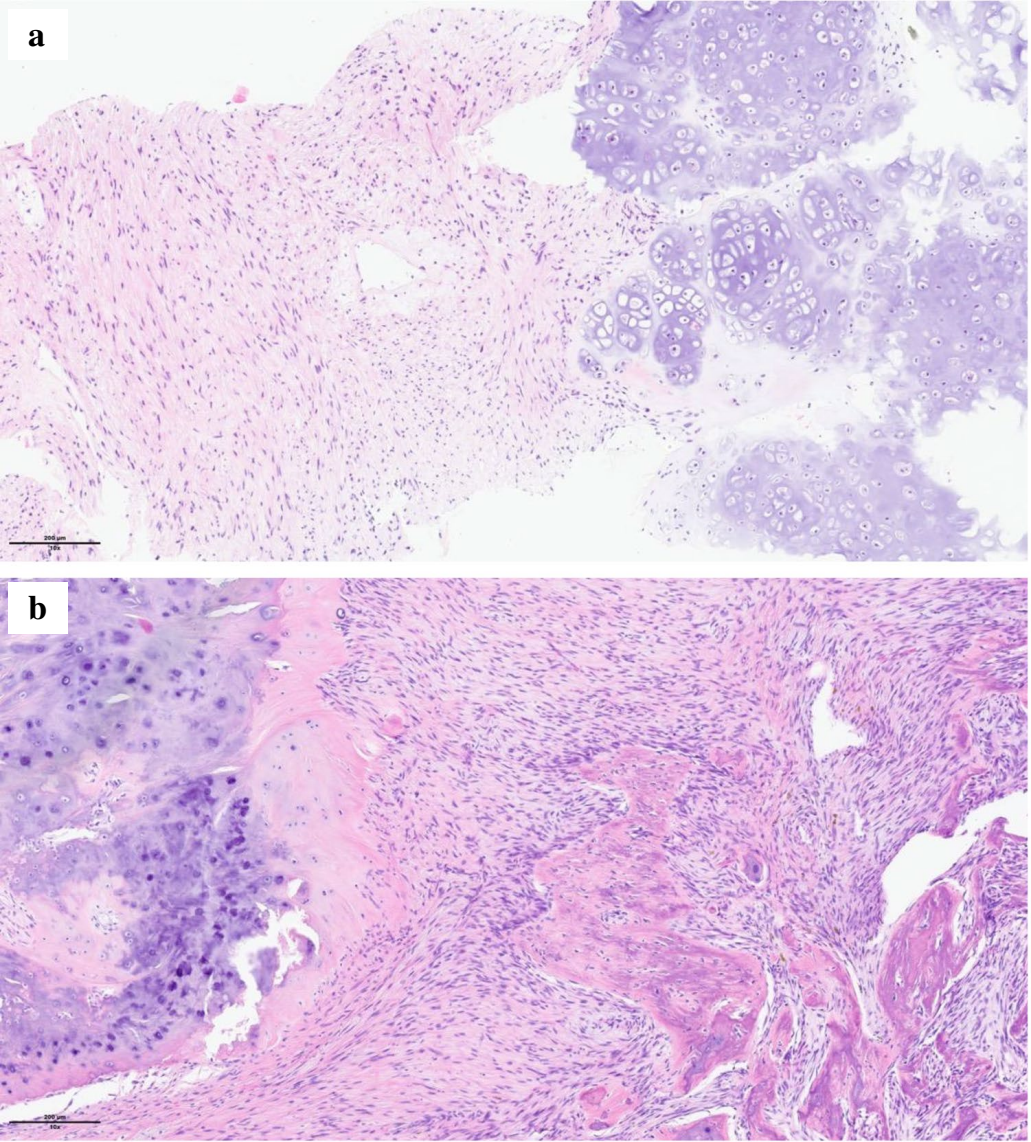
An atypical spindle cell proliferation with zones of chondroid differentiation in the form of well-differentiated, focally hypercellular cartilage characterized by atypical chondrocytes with enlarged, nucleolated nuclei was observed on biopsy (**a** magnification × 10, scale bar 200 µm). The surgical resection specimen showed a dense proliferation of spindle-shaped cells forming short, interlacing bundles. It was associated with trabeculae of woven bone and areas of well-differentiated, hypercellular cartilage containing atypical chondrocytes (**b** magnification × 10, scale bar 200 µm)

However, 24 months after the initiation of denosumab, the radiologic evaluation showed enlargement of the lytic bone lesion as well as its extension in the surrounding soft tissues (Fig. 1h). A CT-guided biopsy of the bone lesion was performed, and the histopathologic analysis showed an atypical spindle cell proliferation with adjacent hyaline cartilage, in keeping with dedifferentiated chondrosarcoma (Fig. 4). The sequencing of a 52-gene panel did not yield additional information, notably no IDH1 and two mutations. Furthermore, no *MYC* amplification was detected by fluorescence in situ hybridization (FISH). The patient underwent an induction chemotherapy according to the EURO-B.O.S.S followed by an en bloc resection. The final hemipelvectomy specimen demonstrated a spindle cell proliferation with chondroblastic differentiation and bone formation, consistent with dedifferentiated chondrosarcoma. The tumor was > 90% viable, in the presence of minimal post-therapeutic changes.

## Discussion

Benign fibrous histiocytoma of bone (BFHB) is a controversial entity characterized by a mixture of fibroblasts arranged in a storiform pattern, varying amounts of osteoclast-type giant cells and foamy macrophages. According to the WHO 2020 classification of bone and soft tissue, this entity is now considered examples of NOF when involving the metaphysis, or GCTB with regressive changes when affecting the epiphysis [11].

In the present case, histopathologic examination demonstrated a proliferation of spindle-shaped cells focally arranged in a storiform pattern with histiocytic foamy cells and numerous multinucleated giant cells. There was no histological sign of malignancy. The initial analysis raised the differential diagnosis of fibrous dysplasia and BFH. Tumor localization, radiology, and absence of *GNAS* mutation reinforced the diagnosis of BFH [12]. Establishing the diagnosis of the recurring bone lesion was challenging. Extensive immunophenotyping and sequencing were conducted. The negativity of mutated *H3-F3A* in immunohistochemistry could not support the diagnosis of GCTB [13, 14]. The involvement of the pelvic bone further supported our initial diagnosis of BFHB.

In the present case, the extension of the tumor to the whole hemipelvis made curative surgical excision virtually impossible, unless extremely mutilating (internal hemipelvectomy), and the tumor recurred within 2 years after both subtotal extensive curettages under O-arm navigation. Therefore, there was a need for an alternative treatment.

Denosumab is a RANKL inhibitor that blocks the RANKL-mediated osteoclast activation and maturation. The FDA initially approved it in 2010 for the prevention of skeletal-related events in bone metastasis of solid tumor. In 2013, it was further approved for the treatment of unresectable GCTB. In their 2013 study, Chawla et al. reported clinical benefit from denosumab with pain reduction in 60% of the GCTB patients. Furthermore, denosumab treatment limited the morbidity of surgery or led to delay and even avoidance of surgery [15]. However, optimal duration of denosumab treatment has yet to be determined. In their recent study, Chawla et al. showed a highly favorable risk to benefice ratio of denosumab, while emphasizing the need of strict monitoring in long-term treated patients exposed to higher risk of osteonecrosis of the jaw [16].

In analogy with the data in GCTB, we hypothesized in the present case that the use of denosumab could prevent progression of disease and bring pain relief. Indeed, our patient showed initial radiologic response and clinical benefit for 24 months. Unfortunately, after 2 years of treatment, the lytic lesion resumed its spread and displayed malignant transformation.

In our patient, the diagnosis of recurrent BFHB has been confirmed on multiple biopsies and curetting specimens, and we observed mineralization of the lytic lesion with clinical improvement under denosumab therapy. Thus, it seems unlikely that the malignant component was present initially and not sampled. Therefore, our patient appeared to have developed a chondrosarcoma in a previously benign, recurrent BFHB.

Of note, cases of GCTB malignant transformation under exclusive denosumab therapy have been reported, mostly into osteosarcoma and undifferentiated pleomorphic sarcoma [17]. Hypocalcemia-induced hyperparathyroidism has been suggested to be responsible for the increased incidence of malignancy in denosumab-treated patient [18]. Yet, the causative role of denosumab has never been formally imputed. Furthermore, in the existing open-label phase II studies, the reported incidence of malignant transformation in denosumab-treated patients is similar to that observed in untreated patients [19–21]. In addition, up to 5% of the patient with GCTB developed secondary malignancy after radiation, with a mean interval from radiation to diagnosis of 10 years and a radiation dose above 40 Gy [22]. To note, no dedifferentiated chondrosarcoma has been reported [22]. In our case, the chondrosarcoma developed only 2 years following the end of the radiotherapy, making it unlikely to be radio-induced. Furthermore, *MYC* amplification, a frequent a genetic alteration of radiation-induced sarcomas, was not detected in this case [23].

The question remains whether it is really a malignant transformation of the primary tumor or a second tumor. Large-scale parallel sequencing of primary and recurrent lesions could confirm filiation between the two tumors. Methylation signatures could also be used to reassure the histopathological diagnosis [24].

## Conclusion

To date, there is no established treatment strategy for patients with relapsing or unresectable benign fibrous histiocytoma of bone. Denosumab is a RANKL inhibitor that has shown very promising clinical activity in giant cell tumors of bone.

Denosumab seems to be a viable option for long-lasting local control of benign fibrous histiocytoma of bone. Yet, close follow-up is required to detect a potential malignant transformation of the tumors under treatment, although more studies are required to assess the role of denosumab in that transformation.

In our case, although denosumab therapy was not curative, it allowed to postpone a mutilating surgery for over two years with a successful pain management and good quality of life.
